# *catena*-Poly[bis­(μ-4-methyl­benzoato-κ^2^ *O*:*O*′)hepta­kis(μ-4-methyl­benzoato-κ^3^ *O*,*O*′:*O*)tris­amarium(III)]

**DOI:** 10.1107/S1600536808014219

**Published:** 2008-05-17

**Authors:** Sun Feng

**Affiliations:** a School of Chemistry and Environment, South China Normal University, Guangzhou 510006, People’s Republic of China

## Abstract

The title samarium coordination polymer, [Sm_3_(C_8_H_7_O_2_)_9_]_*n*_, was obtained by the hydro­thermal reaction of Sm(NO_3_)_3_ with 4-methyl­benzoic acid in alkaline aqueous solution. In the asymmetric unit, there are three crystallographically independent Sm^III^ ions, two of which are eight-coordinate in a distorted square-anti­prismatic environment, while the third is nine-coordinate in a distorted tricapped trigonal-prismatic coordination. The metal centres are coordinated and bridged by four tridentate, three bidentate and two monodentate methyl­benzoate anions, forming polymeric chains running parallel to the *b* axis.

## Related literature

For related literature, see: Li *et al.* (2005 ▶); Jin *et al.* (2001 ▶).
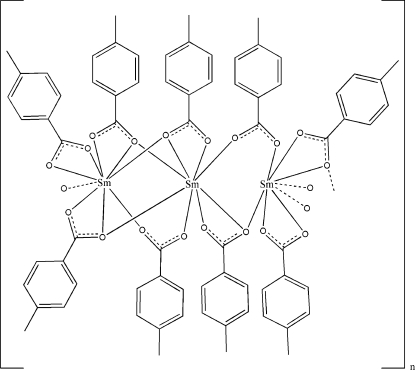


## Experimental

### 

#### Crystal data

[Sm_3_(C_8_H_7_O_2_)_9_]
*M* *_r_* = 1667.27
Monoclinic, 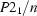

*a* = 13.8527 (2) Å
*b* = 22.5008 (7) Å
*c* = 21.8191 (8) Å
β = 96.580 (3)°
*V* = 6756.1 (3) Å^3^
*Z* = 4
Mo *K*α radiation
μ = 2.64 mm^−1^
*T* = 296 (2) K
0.25 × 0.24 × 0.21 mm

#### Data collection

Bruker APEXII area-detector diffractometer
Absorption correction: multi-scan (*SADABS*; Sheldrick, 1996 ▶) *T*_min_ = 0.525, *T*_max_ = 0.578
57075 measured reflections
12173 independent reflections
9574 reflections with *I* > 2σ(*I*)
*R*_int_ = 0.068

#### Refinement

*R*[*F* ^2^ > 2σ(*F* ^2^)] = 0.037
*wR*(*F* ^2^) = 0.095
*S* = 1.09
12173 reflections
847 parameters
H-atom parameters constrained
Δρ_max_ = 0.61 e Å^−3^
Δρ_min_ = −1.33 e Å^−3^

### 

Data collection: *APEX2* (Bruker, 2004 ▶); cell refinement: *SAINT* (Bruker, 2004 ▶); data reduction: *SAINT*; program(s) used to solve structure: *SHELXS97* (Sheldrick, 2008 ▶); program(s) used to refine structure: *SHELXL97* (Sheldrick, 2008 ▶); molecular graphics: *SHELXTL* (Sheldrick, 2008 ▶); software used to prepare material for publication: *SHELXTL*.

## Supplementary Material

Crystal structure: contains datablocks I, global. DOI: 10.1107/S1600536808014219/rz2201sup1.cif

Structure factors: contains datablocks I. DOI: 10.1107/S1600536808014219/rz2201Isup2.hkl

Additional supplementary materials:  crystallographic information; 3D view; checkCIF report

## Figures and Tables

**Fig. 1.**
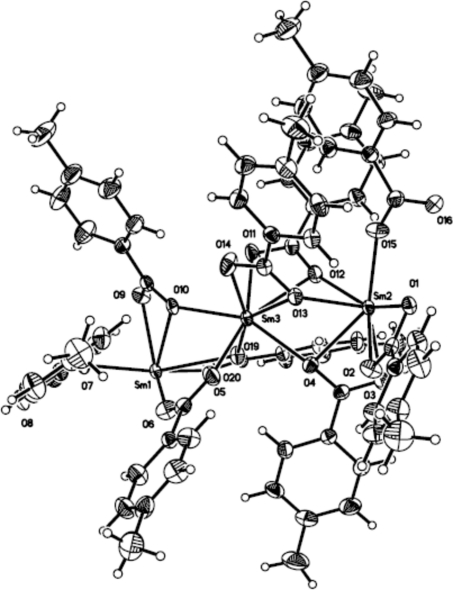
The molecular structure of the title compound showing the atomic-numbering scheme and displacement ellipsoids drawn at the 30% probability level.

## supplementary crystallographic information

### Comment

As a building block, 4-methylbenzoic acid is an excellent candidate for the construction of supramolecular complexes (Jin *et al.*, 2001; Li *et al.*, 2005). Recently, the title coordination polymer was obtained by the reaction of Eu(NO_3_)_3_ with 4-methylbenzoic acid in alkaline aqueous solution, and its crystal structure is reported here.

In the asymmetric unit of the title compound there are three crystallographically independent Sm^III^ ions, two of which are eight-coordinate in a distorted square-antiprismatic environment while the third is nine-coordinate in a distorted tricapped trigonal prismatic coordination (Fig. 1). The Sm1···Sm2, Sm2···Sm3 and Sm1···Sm3^i^ are 3.950 (2), 3.738 (4) and 3.907 (5) Å, respectively (symmetry code: i = 0.5-x, 0.5+y, 0.5-z). The metal centres are bridged by four tridentate, three bidentate and two monodentate methylbenzoato anions to forms polymeric chains running parallel to the b axis .

### Experimental

A mixture of Sm(NO_3_)_3_ (0.5 mmol, 0.168 g), 4-methylbenzoic acid (0.5 mmol, 0.068 g), NaOH (0.5 mmol; 0.02 g) and H_2_O (12 ml) was placed in a 23 ml Teflon reactor, which was heated to 433 K for three days and then cooled to room temperature at a rate of 10 K h^-1^. The crystals obtained were washed with water and dryed in air.

### Refinement

Carbon-bound H atoms were placed at calculated positions and were treated as riding on the parent C atoms with C—H = 0.93–0.96 Å, and with *U*_iso_(H) = 1.2 *U*_eq_(C) or 1.5 *U*_eq_(C) for methyl H atoms.

### Crystal data

**Table:**

[Sm_3_(C_8_H_7_O_2_)_9_]	*F*_000_ = 3300
*M**_r_* = 1667.27	*D*_x_ = 1.639 Mg m^−^^3^
Monoclinic, *P*2_1_/*n*	Mo *K*α radiation λ = 0.71073 Å
Hall symbol: -P 2yn	Cell parameters from 8289 reflections
*a* = 13.8527 (2) Å	θ = 1.7–28.0º
*b* = 22.5008 (7) Å	µ = 2.64 mm^−^^1^
*c* = 21.8191 (8) Å	*T* = 296 (2) K
β = 96.580 (3)º	Block, colorless
*V* = 6756.1 (3) Å^3^	0.25 × 0.24 × 0.21 mm
*Z* = 4	

### Data collection

**Table:**

Bruker APEXII area-detector diffractometer	12173 independent reflections
Radiation source: fine-focus sealed tube	9574 reflections with *I* > 2σ(*I*)
Monochromator: graphite	*R*_int_ = 0.068
*T* = 296(2) K	θ_max_ = 25.2º
φ and ω scans	θ_min_ = 1.3º
Absorption correction: multi-scan(SADABS; Sheldrick, 1996)	*h* = −16→16
*T*_min_ = 0.525, *T*_max_ = 0.578	*k* = −26→26
57075 measured reflections	*l* = −26→24

### Refinement

**Table:**

Refinement on *F*^2^	Secondary atom site location: difference Fourier map
Least-squares matrix: full	Hydrogen site location: inferred from neighbouring sites
*R*[*F*^2^ > 2σ(*F*^2^)] = 0.037	H-atom parameters constrained
*wR*(*F*^2^) = 0.095	*w* = 1/[σ^2^(*F*_o_^2^) + (0.0378*P*)^2^ + 5.7051*P*] where *P* = (*F*_o_^2^ + 2*F*_c_^2^)/3
*S* = 1.09	(Δ/σ)_max_ = 0.002
12173 reflections	Δρ_max_ = 0.61 e Å^−^^3^
847 parameters	Δρ_min_ = −1.33 e Å^−^^3^
Primary atom site location: structure-invariant direct methods	Extinction correction: none

### Special details

**Table:**

Geometry. All e.s.d.'s (except the e.s.d. in the dihedral angle between two l.s. planes) are estimated using the full covariance matrix. The cell e.s.d.'s are taken into account individually in the estimation of e.s.d.'s in distances, angles and torsion angles; correlations between e.s.d.'s in cell parameters are only used when they are defined by crystal symmetry. An approximate (isotropic) treatment of cell e.s.d.'s is used for estimating e.s.d.'s involving l.s. planes.
Refinement. Refinement of *F*^2^ against ALL reflections. The weighted *R*-factor *wR* and goodness of fit *S* are based on *F*^2^, conventional *R*-factors *R* are based on *F*, with *F* set to zero for negative *F*^2^. The threshold expression of *F*^2^ > σ(*F*^2^) is used only for calculating *R*-factors(gt) *etc*. and is not relevant to the choice of reflections for refinement. *R*-factors based on *F*^2^ are statistically about twice as large as those based on *F*, and *R*- factors based on ALL data will be even larger.

### Fractional atomic coordinates and isotropic or equivalent isotropic displacement parameters (Å^2^)

**Table:**

	*x*	*y*	*z*	*U*_iso_*/*U*_eq_	
C1	0.3049 (4)	0.4259 (2)	0.1406 (2)	0.0367 (12)	
C2	0.3259 (4)	0.4090 (2)	0.0775 (2)	0.0392 (12)	
C3	0.3955 (4)	0.4401 (3)	0.0509 (3)	0.0528 (15)	
H3	0.4293	0.4707	0.0725	0.063*	
C4	0.4155 (5)	0.4259 (3)	−0.0085 (3)	0.0635 (18)	
H4	0.4631	0.4472	−0.0259	0.076*	
C5	0.3672 (5)	0.3816 (3)	−0.0419 (3)	0.0584 (17)	
C6	0.2992 (6)	0.3506 (3)	−0.0142 (3)	0.0660 (19)	
H6	0.2664	0.3196	−0.0358	0.079*	
C7	0.2772 (5)	0.3635 (3)	0.0448 (2)	0.0499 (15)	
H7	0.2302	0.3417	0.0621	0.060*	
C8	0.3895 (7)	0.3672 (4)	−0.1069 (3)	0.088 (3)	
H8A	0.4098	0.3265	−0.1087	0.132*	
H8B	0.4405	0.3927	−0.1176	0.132*	
H8C	0.3322	0.3733	−0.1354	0.132*	
C9	0.4184 (4)	0.5513 (2)	0.2748 (2)	0.0363 (12)	
C10	0.5085 (4)	0.5868 (2)	0.2799 (2)	0.0364 (12)	
C11	0.5969 (4)	0.5606 (3)	0.3028 (3)	0.0520 (16)	
H11	0.5989	0.5204	0.3130	0.062*	
C12	0.6806 (5)	0.5936 (3)	0.3102 (3)	0.0655 (19)	
H12	0.7386	0.5757	0.3262	0.079*	
C13	0.6805 (5)	0.6534 (3)	0.2944 (3)	0.0589 (17)	
C14	0.5941 (4)	0.6786 (3)	0.2706 (3)	0.0558 (16)	
H14	0.5925	0.7184	0.2591	0.067*	
C15	0.5100 (4)	0.6460 (2)	0.2637 (3)	0.0463 (14)	
H15	0.4524	0.6643	0.2476	0.056*	
C16	0.7733 (5)	0.6894 (4)	0.3053 (4)	0.097 (3)	
H16A	0.7642	0.7270	0.2845	0.146*	
H16B	0.8252	0.6682	0.2894	0.146*	
H16C	0.7894	0.6960	0.3487	0.146*	
C17	0.3320 (4)	0.7272 (3)	0.1541 (3)	0.0441 (14)	
C18	0.3746 (4)	0.7327 (2)	0.0949 (2)	0.0371 (12)	
C19	0.4264 (5)	0.7827 (3)	0.0816 (3)	0.0556 (16)	
H19	0.4340	0.8139	0.1097	0.067*	
C20	0.4672 (5)	0.7866 (3)	0.0263 (3)	0.071 (2)	
H20	0.5012	0.8206	0.0176	0.085*	
C21	0.4578 (5)	0.7409 (3)	−0.0156 (3)	0.0611 (18)	
C22	0.4053 (5)	0.6921 (3)	−0.0019 (3)	0.0665 (19)	
H22	0.3970	0.6611	−0.0301	0.080*	
C23	0.3645 (5)	0.6876 (3)	0.0524 (3)	0.0578 (17)	
H23	0.3297	0.6537	0.0604	0.069*	
C24	0.5035 (7)	0.7440 (4)	−0.0752 (3)	0.098 (3)	
H24A	0.5306	0.7060	−0.0836	0.148*	
H24B	0.5540	0.7734	−0.0715	0.148*	
H24C	0.4549	0.7546	−0.1084	0.148*	
C25	0.2101 (4)	0.8949 (2)	0.1485 (2)	0.0343 (12)	
C26	0.1816 (4)	0.8575 (2)	0.0936 (2)	0.0375 (12)	
C27	0.1914 (5)	0.8789 (3)	0.0356 (3)	0.0568 (17)	
H27	0.2148	0.9172	0.0311	0.068*	
C28	0.1670 (6)	0.8441 (3)	−0.0159 (3)	0.068 (2)	
H28	0.1727	0.8597	−0.0548	0.082*	
C29	0.1342 (5)	0.7869 (3)	−0.0105 (3)	0.0539 (16)	
C30	0.1219 (5)	0.7658 (3)	0.0474 (3)	0.0523 (16)	
H30	0.0973	0.7278	0.0517	0.063*	
C31	0.1457 (4)	0.8007 (2)	0.0991 (2)	0.0390 (13)	
H31	0.1375	0.7857	0.1379	0.047*	
C32	0.1106 (6)	0.7477 (3)	−0.0677 (3)	0.082 (2)	
H32A	0.0977	0.7724	−0.1036	0.123*	
H32B	0.0544	0.7239	−0.0630	0.123*	
H32C	0.1649	0.7223	−0.0724	0.123*	
C33	0.0824 (4)	0.7759 (2)	0.2579 (2)	0.0364 (12)	
C34	−0.0177 (4)	0.7831 (2)	0.2281 (2)	0.0404 (13)	
C35	−0.0660 (5)	0.8349 (3)	0.2368 (3)	0.069 (2)	
H35	−0.0364	0.8640	0.2628	0.083*	
C36	−0.1578 (6)	0.8444 (4)	0.2074 (4)	0.080 (2)	
H36	−0.1894	0.8800	0.2134	0.096*	
C37	−0.2038 (5)	0.8018 (4)	0.1691 (4)	0.076 (2)	
C38	−0.1543 (5)	0.7502 (4)	0.1608 (3)	0.075 (2)	
H38	−0.1843	0.7207	0.1355	0.089*	
C39	−0.0619 (4)	0.7408 (3)	0.1889 (3)	0.0566 (16)	
H39	−0.0292	0.7059	0.1814	0.068*	
C40	−0.3065 (6)	0.8119 (5)	0.1373 (5)	0.115 (4)	
H40A	−0.3067	0.8079	0.0935	0.172*	
H40B	−0.3278	0.8512	0.1467	0.172*	
H40C	−0.3497	0.7831	0.1518	0.172*	
C41	0.0982 (4)	0.5836 (2)	0.3264 (2)	0.0398 (13)	
C42	0.0341 (4)	0.5525 (2)	0.3654 (2)	0.0381 (12)	
C43	−0.0362 (5)	0.5844 (3)	0.3920 (3)	0.0587 (17)	
H43	−0.0412	0.6253	0.3862	0.070*	
C44	−0.0984 (5)	0.5558 (4)	0.4267 (3)	0.070 (2)	
H44	−0.1452	0.5778	0.4441	0.083*	
C45	−0.0933 (5)	0.4955 (3)	0.4364 (3)	0.0557 (16)	
C46	−0.0223 (5)	0.4639 (3)	0.4109 (3)	0.0613 (18)	
H46	−0.0170	0.4231	0.4172	0.074*	
C47	0.0409 (5)	0.4924 (3)	0.3760 (3)	0.0496 (15)	
H47	0.0887	0.4706	0.3594	0.059*	
C48	−0.1626 (5)	0.4639 (4)	0.4741 (3)	0.081 (2)	
H48A	−0.1894	0.4920	0.5007	0.121*	
H48B	−0.1283	0.4335	0.4986	0.121*	
H48C	−0.2142	0.4463	0.4470	0.121*	
C49	0.0347 (4)	0.3963 (3)	0.2248 (3)	0.0450 (14)	
C50	−0.0703 (4)	0.4102 (2)	0.2115 (2)	0.0395 (12)	
C51	−0.1052 (5)	0.4659 (3)	0.2248 (3)	0.0537 (16)	
H51	−0.0645	0.4943	0.2452	0.064*	
C52	−0.2030 (5)	0.4785 (3)	0.2067 (3)	0.0667 (19)	
H52	−0.2274	0.5154	0.2162	0.080*	
C53	−0.2641 (5)	0.4381 (3)	0.1756 (3)	0.0611 (18)	
C54	−0.2284 (5)	0.3826 (3)	0.1648 (3)	0.0628 (18)	
H54	−0.2699	0.3540	0.1455	0.075*	
C55	−0.1324 (4)	0.3685 (3)	0.1820 (3)	0.0484 (14)	
H55	−0.1094	0.3309	0.1737	0.058*	
C56	−0.3693 (5)	0.4546 (4)	0.1534 (4)	0.103 (3)	
H56A	−0.3995	0.4709	0.1872	0.155*	
H56B	−0.4040	0.4197	0.1382	0.155*	
H56C	−0.3705	0.4836	0.1210	0.155*	
C57	0.0873 (4)	0.5670 (2)	0.1536 (2)	0.0366 (12)	
C58	0.0181 (4)	0.5315 (2)	0.1118 (2)	0.0381 (12)	
C59	−0.0730 (4)	0.5542 (3)	0.0921 (3)	0.0505 (15)	
H59	−0.0890	0.5924	0.1037	0.061*	
C60	−0.1402 (5)	0.5209 (3)	0.0556 (3)	0.0551 (16)	
H60	−0.2014	0.5366	0.0431	0.066*	
C61	−0.1177 (5)	0.4643 (3)	0.0373 (3)	0.0517 (16)	
C62	−0.0264 (5)	0.4420 (3)	0.0558 (3)	0.0525 (16)	
H62	−0.0105	0.4041	0.0432	0.063*	
C63	0.0417 (4)	0.4747 (2)	0.0924 (2)	0.0426 (13)	
H63	0.1031	0.4590	0.1041	0.051*	
C64	−0.1926 (5)	0.4270 (3)	−0.0016 (3)	0.076 (2)	
H64A	−0.1601	0.3973	−0.0233	0.114*	
H64B	−0.2305	0.4521	−0.0308	0.114*	
H64C	−0.2344	0.4081	0.0246	0.114*	
C65	0.3291 (4)	0.7149 (2)	0.3703 (2)	0.0337 (11)	
C66	0.3513 (4)	0.6720 (2)	0.4208 (2)	0.0362 (12)	
C67	0.3792 (5)	0.6917 (3)	0.4804 (3)	0.0550 (16)	
H67	0.3812	0.7323	0.4885	0.066*	
C68	0.4040 (5)	0.6526 (3)	0.5275 (3)	0.0567 (17)	
H68	0.4216	0.6670	0.5672	0.068*	
C69	0.4034 (4)	0.5920 (3)	0.5173 (3)	0.0467 (14)	
C70	0.3746 (4)	0.5723 (3)	0.4583 (3)	0.0509 (15)	
H70	0.3729	0.5316	0.4504	0.061*	
C71	0.3484 (4)	0.6113 (2)	0.4109 (3)	0.0474 (14)	
H71	0.3284	0.5968	0.3716	0.057*	
C72	0.4347 (6)	0.5499 (3)	0.5700 (3)	0.074 (2)	
H72A	0.5038	0.5524	0.5803	0.111*	
H72B	0.4172	0.5100	0.5578	0.111*	
H72C	0.4030	0.5607	0.6053	0.111*	
Sm2	0.204869 (19)	0.640988 (10)	0.239154 (11)	0.02989 (8)	
Sm3	0.247655 (18)	0.477166 (10)	0.251356 (11)	0.02855 (8)	
Sm1	0.282165 (18)	0.806693 (10)	0.279308 (11)	0.02956 (8)	
O1	0.2392 (3)	0.39776 (15)	0.16682 (15)	0.0368 (8)	
O2	0.3486 (3)	0.46826 (18)	0.16786 (18)	0.0512 (10)	
O3	0.4185 (3)	0.49858 (17)	0.29158 (18)	0.0479 (10)	
O4	0.3385 (2)	0.57567 (15)	0.25193 (16)	0.0382 (8)	
O5	0.2850 (3)	0.68050 (18)	0.16290 (19)	0.0556 (11)	
O6	0.3440 (3)	0.7683 (2)	0.19299 (18)	0.0551 (11)	
O7	0.2092 (3)	0.87445 (14)	0.20322 (14)	0.0347 (8)	
O8	0.2347 (3)	0.94762 (16)	0.14133 (16)	0.0446 (9)	
O9	0.1154 (3)	0.80769 (16)	0.30229 (17)	0.0452 (9)	
O10	0.1397 (2)	0.73852 (14)	0.23482 (15)	0.0341 (8)	
O11	0.0900 (3)	0.63750 (16)	0.3154 (2)	0.0597 (12)	
O12	0.1641 (3)	0.55398 (16)	0.30265 (17)	0.0424 (9)	
O13	0.1684 (2)	0.54455 (15)	0.17555 (16)	0.0375 (8)	
O14	0.0667 (3)	0.61854 (17)	0.1674 (2)	0.0615 (12)	
O15	0.0931 (3)	0.4370 (2)	0.24686 (18)	0.0539 (11)	
O16	0.0624 (3)	0.34545 (19)	0.2123 (2)	0.0600 (12)	
O19	0.3002 (3)	0.69764 (15)	0.31549 (15)	0.0384 (8)	
O20	0.3381 (3)	0.76940 (16)	0.37995 (17)	0.0567 (12)	

### Atomic displacement parameters (Å^2^)

**Table:**

	*U*^11^	*U*^22^	*U*^33^	*U*^12^	*U*^13^	*U*^23^
C1	0.038 (3)	0.033 (3)	0.038 (3)	0.010 (2)	−0.001 (2)	0.001 (2)
C2	0.041 (3)	0.040 (3)	0.036 (3)	0.005 (2)	0.004 (2)	0.006 (2)
C3	0.053 (4)	0.059 (4)	0.048 (4)	−0.007 (3)	0.009 (3)	−0.006 (3)
C4	0.056 (4)	0.087 (5)	0.051 (4)	−0.003 (4)	0.020 (3)	−0.003 (4)
C5	0.072 (4)	0.065 (4)	0.039 (3)	0.006 (4)	0.012 (3)	−0.005 (3)
C6	0.101 (6)	0.057 (4)	0.038 (4)	−0.008 (4)	0.000 (4)	−0.010 (3)
C7	0.072 (4)	0.046 (3)	0.033 (3)	−0.003 (3)	0.009 (3)	−0.002 (3)
C8	0.117 (7)	0.104 (7)	0.047 (4)	0.010 (5)	0.028 (4)	−0.013 (4)
C9	0.034 (3)	0.039 (3)	0.036 (3)	0.001 (2)	0.004 (2)	−0.005 (2)
C10	0.037 (3)	0.041 (3)	0.031 (3)	0.001 (2)	0.005 (2)	−0.001 (2)
C11	0.043 (3)	0.052 (4)	0.059 (4)	0.004 (3)	−0.004 (3)	0.008 (3)
C12	0.039 (3)	0.084 (5)	0.071 (5)	−0.006 (3)	−0.005 (3)	0.003 (4)
C13	0.048 (4)	0.078 (5)	0.051 (4)	−0.019 (3)	0.007 (3)	0.000 (3)
C14	0.049 (4)	0.050 (4)	0.068 (4)	−0.018 (3)	0.006 (3)	0.003 (3)
C15	0.048 (3)	0.040 (3)	0.050 (3)	0.000 (3)	0.002 (3)	0.005 (3)
C16	0.058 (5)	0.118 (8)	0.114 (7)	−0.037 (5)	0.005 (4)	0.012 (6)
C17	0.043 (3)	0.050 (4)	0.039 (3)	0.012 (3)	0.007 (3)	0.003 (3)
C18	0.041 (3)	0.033 (3)	0.038 (3)	0.000 (2)	0.008 (2)	0.000 (2)
C19	0.073 (4)	0.045 (4)	0.051 (4)	−0.005 (3)	0.016 (3)	−0.008 (3)
C20	0.085 (5)	0.057 (4)	0.078 (5)	0.002 (4)	0.038 (4)	0.015 (4)
C21	0.072 (5)	0.064 (5)	0.051 (4)	0.027 (4)	0.021 (3)	0.005 (3)
C22	0.080 (5)	0.078 (5)	0.043 (4)	0.004 (4)	0.017 (3)	−0.017 (3)
C23	0.061 (4)	0.059 (4)	0.054 (4)	−0.010 (3)	0.009 (3)	−0.016 (3)
C24	0.130 (8)	0.109 (7)	0.066 (5)	0.043 (6)	0.052 (5)	0.020 (5)
C25	0.034 (3)	0.032 (3)	0.037 (3)	0.004 (2)	0.003 (2)	−0.002 (2)
C26	0.045 (3)	0.038 (3)	0.029 (3)	0.000 (2)	0.000 (2)	0.002 (2)
C27	0.083 (5)	0.048 (4)	0.041 (3)	−0.019 (3)	0.015 (3)	0.002 (3)
C28	0.105 (6)	0.072 (5)	0.028 (3)	−0.007 (4)	0.011 (3)	−0.001 (3)
C29	0.067 (4)	0.055 (4)	0.039 (3)	0.000 (3)	0.001 (3)	−0.009 (3)
C30	0.066 (4)	0.038 (3)	0.049 (4)	−0.004 (3)	−0.012 (3)	−0.005 (3)
C31	0.049 (3)	0.041 (3)	0.025 (3)	−0.001 (3)	0.000 (2)	−0.002 (2)
C32	0.121 (7)	0.082 (6)	0.042 (4)	−0.015 (5)	0.002 (4)	−0.024 (4)
C33	0.045 (3)	0.025 (3)	0.040 (3)	−0.001 (2)	0.010 (2)	0.003 (2)
C34	0.040 (3)	0.039 (3)	0.043 (3)	0.004 (2)	0.009 (2)	−0.002 (3)
C35	0.071 (5)	0.070 (5)	0.066 (5)	0.024 (4)	0.005 (4)	−0.022 (4)
C36	0.075 (5)	0.084 (6)	0.080 (5)	0.042 (5)	0.007 (4)	−0.001 (5)
C37	0.051 (4)	0.106 (7)	0.070 (5)	0.021 (4)	0.000 (4)	0.023 (5)
C38	0.055 (4)	0.083 (6)	0.080 (5)	0.001 (4)	−0.015 (4)	−0.002 (4)
C39	0.047 (4)	0.050 (4)	0.071 (4)	0.007 (3)	−0.003 (3)	−0.001 (3)
C40	0.061 (5)	0.141 (9)	0.134 (8)	0.032 (5)	−0.026 (5)	0.011 (7)
C41	0.048 (3)	0.032 (3)	0.039 (3)	0.002 (2)	0.003 (3)	−0.001 (2)
C42	0.043 (3)	0.037 (3)	0.036 (3)	−0.001 (2)	0.008 (2)	−0.002 (2)
C43	0.058 (4)	0.054 (4)	0.067 (4)	0.010 (3)	0.022 (3)	0.005 (3)
C44	0.056 (4)	0.085 (6)	0.074 (5)	0.012 (4)	0.033 (4)	0.007 (4)
C45	0.052 (4)	0.073 (5)	0.044 (4)	−0.015 (3)	0.013 (3)	0.003 (3)
C46	0.072 (5)	0.051 (4)	0.065 (4)	−0.013 (3)	0.025 (4)	0.008 (3)
C47	0.063 (4)	0.042 (3)	0.048 (4)	−0.002 (3)	0.026 (3)	−0.001 (3)
C48	0.067 (5)	0.108 (7)	0.073 (5)	−0.024 (4)	0.032 (4)	0.004 (5)
C49	0.038 (3)	0.051 (4)	0.046 (3)	0.004 (3)	0.006 (3)	0.014 (3)
C50	0.039 (3)	0.043 (3)	0.037 (3)	0.001 (2)	0.008 (2)	0.005 (2)
C51	0.063 (4)	0.039 (3)	0.060 (4)	0.000 (3)	0.010 (3)	−0.005 (3)
C52	0.067 (5)	0.051 (4)	0.084 (5)	0.021 (3)	0.019 (4)	0.004 (4)
C53	0.045 (4)	0.080 (5)	0.058 (4)	0.006 (4)	0.003 (3)	0.018 (4)
C54	0.046 (4)	0.074 (5)	0.066 (4)	−0.007 (3)	−0.004 (3)	−0.001 (4)
C55	0.034 (3)	0.051 (4)	0.060 (4)	0.000 (3)	0.003 (3)	−0.008 (3)
C56	0.052 (5)	0.132 (8)	0.124 (8)	0.019 (5)	0.000 (5)	0.040 (6)
C57	0.042 (3)	0.034 (3)	0.034 (3)	0.001 (2)	0.004 (2)	0.000 (2)
C58	0.041 (3)	0.034 (3)	0.038 (3)	−0.007 (2)	0.000 (2)	0.000 (2)
C59	0.049 (3)	0.046 (4)	0.054 (4)	0.003 (3)	−0.005 (3)	−0.006 (3)
C60	0.044 (3)	0.058 (4)	0.060 (4)	−0.002 (3)	−0.008 (3)	−0.003 (3)
C61	0.061 (4)	0.057 (4)	0.035 (3)	−0.014 (3)	−0.004 (3)	0.002 (3)
C62	0.074 (4)	0.033 (3)	0.049 (4)	−0.012 (3)	−0.003 (3)	−0.006 (3)
C63	0.050 (3)	0.038 (3)	0.039 (3)	0.000 (3)	0.001 (3)	0.000 (2)
C64	0.074 (5)	0.085 (5)	0.063 (5)	−0.033 (4)	−0.015 (4)	−0.013 (4)
C65	0.038 (3)	0.029 (3)	0.034 (3)	0.001 (2)	0.002 (2)	0.002 (2)
C66	0.041 (3)	0.032 (3)	0.035 (3)	−0.001 (2)	0.003 (2)	0.003 (2)
C67	0.082 (5)	0.041 (3)	0.039 (3)	−0.003 (3)	−0.005 (3)	0.001 (3)
C68	0.068 (4)	0.070 (5)	0.030 (3)	−0.005 (3)	−0.004 (3)	0.005 (3)
C69	0.044 (3)	0.050 (4)	0.047 (4)	0.002 (3)	0.004 (3)	0.020 (3)
C70	0.061 (4)	0.032 (3)	0.059 (4)	0.009 (3)	0.003 (3)	0.010 (3)
C71	0.060 (4)	0.039 (3)	0.043 (3)	0.008 (3)	0.004 (3)	0.002 (3)
C72	0.080 (5)	0.077 (5)	0.067 (5)	0.013 (4)	0.012 (4)	0.037 (4)
Sm2	0.03705 (15)	0.01945 (13)	0.03285 (15)	−0.00120 (10)	0.00262 (11)	−0.00084 (10)
Sm3	0.03357 (15)	0.02041 (13)	0.03115 (15)	−0.00017 (10)	0.00149 (11)	−0.00065 (10)
Sm1	0.03868 (15)	0.02074 (13)	0.02891 (15)	−0.00141 (10)	0.00231 (11)	0.00089 (10)
O1	0.047 (2)	0.0297 (19)	0.034 (2)	0.0015 (16)	0.0071 (16)	0.0049 (15)
O2	0.058 (3)	0.052 (3)	0.047 (2)	−0.016 (2)	0.020 (2)	−0.016 (2)
O3	0.045 (2)	0.035 (2)	0.060 (3)	−0.0036 (18)	−0.0052 (19)	0.0092 (19)
O4	0.0347 (19)	0.036 (2)	0.042 (2)	0.0013 (16)	−0.0015 (16)	−0.0049 (16)
O5	0.065 (3)	0.047 (3)	0.058 (3)	0.000 (2)	0.022 (2)	0.013 (2)
O6	0.055 (3)	0.068 (3)	0.043 (2)	0.003 (2)	0.0087 (19)	−0.013 (2)
O7	0.051 (2)	0.0289 (18)	0.0236 (18)	0.0034 (16)	0.0018 (15)	0.0022 (14)
O8	0.069 (3)	0.030 (2)	0.035 (2)	−0.0079 (18)	0.0060 (18)	0.0006 (16)
O9	0.051 (2)	0.044 (2)	0.042 (2)	−0.0090 (18)	0.0107 (18)	−0.0107 (18)
O10	0.046 (2)	0.0185 (17)	0.039 (2)	−0.0014 (15)	0.0061 (16)	0.0007 (14)
O11	0.077 (3)	0.027 (2)	0.083 (3)	0.010 (2)	0.043 (2)	0.007 (2)
O12	0.056 (2)	0.032 (2)	0.044 (2)	0.0071 (18)	0.0234 (18)	0.0045 (16)
O13	0.041 (2)	0.0316 (19)	0.039 (2)	−0.0005 (16)	−0.0014 (16)	0.0017 (16)
O14	0.062 (3)	0.034 (2)	0.082 (3)	0.009 (2)	−0.024 (2)	−0.020 (2)
O15	0.039 (2)	0.072 (3)	0.050 (2)	−0.016 (2)	0.0040 (18)	−0.006 (2)
O16	0.040 (2)	0.051 (3)	0.089 (3)	0.007 (2)	0.007 (2)	0.014 (2)
O19	0.052 (2)	0.033 (2)	0.0278 (19)	0.0024 (17)	−0.0038 (16)	−0.0015 (15)
O20	0.104 (3)	0.024 (2)	0.037 (2)	−0.001 (2)	−0.012 (2)	0.0002 (16)

### Geometric parameters (Å, °)

**Table:**

C1—O2	1.244 (6)	C44—C45	1.374 (10)
C1—O1	1.295 (6)	C44—H44	0.9300
C1—C2	1.487 (7)	C45—C46	1.382 (9)
C1—Sm3	2.871 (5)	C45—C48	1.511 (9)
C2—C3	1.373 (8)	C46—C47	1.381 (8)
C2—C7	1.379 (8)	C46—H46	0.9300
C3—C4	1.394 (8)	C47—H47	0.9300
C3—H3	0.9300	C48—H48A	0.9600
C4—C5	1.364 (9)	C48—H48B	0.9600
C4—H4	0.9300	C48—H48C	0.9600
C5—C6	1.366 (10)	C49—O16	1.247 (7)
C5—C8	1.521 (9)	C49—O15	1.278 (7)
C6—C7	1.387 (8)	C49—C50	1.484 (7)
C6—H6	0.9300	C50—C55	1.381 (7)
C7—H7	0.9300	C50—C51	1.387 (8)
C8—H8A	0.9600	C51—C52	1.395 (9)
C8—H8B	0.9600	C51—H51	0.9300
C8—H8C	0.9600	C52—C53	1.367 (10)
C9—O3	1.240 (6)	C52—H52	0.9300
C9—O4	1.284 (6)	C53—C54	1.374 (10)
C9—C10	1.475 (7)	C53—C56	1.527 (9)
C9—Sm3	2.891 (5)	C54—C55	1.377 (8)
C10—C15	1.379 (7)	C54—H54	0.9300
C10—C11	1.399 (7)	C55—H55	0.9300
C11—C12	1.372 (9)	C56—H56A	0.9600
C11—H11	0.9300	C56—H56B	0.9600
C12—C13	1.388 (10)	C56—H56C	0.9600
C12—H12	0.9300	C57—O14	1.240 (6)
C13—C14	1.371 (9)	C57—O13	1.274 (6)
C13—C16	1.516 (9)	C57—C58	1.479 (7)
C14—C15	1.369 (8)	C57—Sm2	2.866 (5)
C14—H14	0.9300	C58—C59	1.383 (8)
C15—H15	0.9300	C58—C63	1.397 (7)
C16—H16A	0.9600	C59—C60	1.376 (8)
C16—H16B	0.9600	C59—H59	0.9300
C16—H16C	0.9600	C60—C61	1.379 (8)
C17—O6	1.253 (7)	C60—H60	0.9300
C17—O5	1.262 (7)	C61—C62	1.377 (9)
C17—C18	1.485 (8)	C61—C64	1.516 (8)
C18—C23	1.371 (8)	C62—C63	1.377 (8)
C18—C19	1.383 (8)	C62—H62	0.9300
C19—C20	1.393 (9)	C63—H63	0.9300
C19—H19	0.9300	C64—H64A	0.9600
C20—C21	1.372 (10)	C64—H64B	0.9600
C20—H20	0.9300	C64—H64C	0.9600
C21—C22	1.369 (10)	C65—O20	1.248 (6)
C21—C24	1.512 (9)	C65—O19	1.277 (6)
C22—C23	1.373 (9)	C65—C66	1.472 (7)
C22—H22	0.9300	C65—Sm1	2.887 (5)
C23—H23	0.9300	C66—C71	1.382 (7)
C24—H24A	0.9600	C66—C67	1.386 (7)
C24—H24B	0.9600	C67—C68	1.367 (8)
C24—H24C	0.9600	C67—H67	0.9300
C25—O8	1.249 (6)	C68—C69	1.382 (8)
C25—O7	1.281 (6)	C68—H68	0.9300
C25—C26	1.479 (7)	C69—C70	1.377 (8)
C25—Sm3^i^	2.873 (5)	C69—C72	1.513 (8)
C26—C27	1.375 (7)	C70—C71	1.375 (7)
C26—C31	1.382 (7)	C70—H70	0.9300
C27—C28	1.378 (8)	C71—H71	0.9300
C27—H27	0.9300	C72—H72A	0.9600
C28—C29	1.375 (9)	C72—H72B	0.9600
C28—H28	0.9300	C72—H72C	0.9600
C29—C30	1.378 (8)	Sm2—O5	2.284 (4)
C29—C32	1.532 (8)	Sm2—O4	2.355 (3)
C30—C31	1.383 (7)	Sm2—O10	2.371 (3)
C30—H30	0.9300	Sm2—O19	2.375 (3)
C31—H31	0.9300	Sm2—O14	2.385 (4)
C32—H32A	0.9600	Sm2—O11	2.432 (4)
C32—H32B	0.9600	Sm2—O12	2.500 (3)
C32—H32C	0.9600	Sm2—O13	2.594 (3)
C33—O9	1.248 (6)	Sm2—Sm3	3.7381 (3)
C33—O10	1.297 (6)	Sm2—Sm1	3.9498 (3)
C33—C34	1.471 (7)	Sm3—O15	2.316 (4)
C33—Sm1	2.839 (5)	Sm3—O13	2.413 (3)
C34—C35	1.368 (8)	Sm3—O8^ii^	2.419 (4)
C34—C39	1.376 (8)	Sm3—O12	2.424 (4)
C35—C36	1.374 (10)	Sm3—O2	2.429 (4)
C35—H35	0.9300	Sm3—O3	2.476 (4)
C36—C37	1.378 (11)	Sm3—O4	2.548 (3)
C36—H36	0.9300	Sm3—O7^ii^	2.558 (3)
C37—C38	1.371 (10)	Sm3—O1	2.561 (3)
C37—C40	1.527 (10)	Sm3—C25^ii^	2.873 (5)
C38—C39	1.371 (9)	Sm1—O16^i^	2.310 (4)
C38—H38	0.9300	Sm1—O6	2.324 (4)
C39—H39	0.9300	Sm1—O7	2.391 (3)
C40—H40A	0.9600	Sm1—O20	2.395 (4)
C40—H40B	0.9600	Sm1—O1^i^	2.398 (3)
C40—H40C	0.9600	Sm1—O9	2.419 (4)
C41—O11	1.238 (6)	Sm1—O19	2.581 (3)
C41—O12	1.286 (6)	Sm1—O10	2.599 (3)
C41—C42	1.476 (8)	Sm1—Sm3^i^	3.9075 (3)
C41—Sm2	2.848 (6)	O1—Sm1^ii^	2.398 (3)
C42—C47	1.374 (8)	O7—Sm3^i^	2.558 (3)
C42—C43	1.389 (8)	O8—Sm3^i^	2.419 (4)
C43—C44	1.370 (9)	O16—Sm1^ii^	2.310 (4)
C43—H43	0.9300		
			
O2—C1—O1	119.4 (5)	C67—C68—H68	119.4
O2—C1—C2	120.2 (5)	C69—C68—H68	119.4
O1—C1—C2	120.4 (5)	C70—C69—C68	117.7 (5)
O2—C1—Sm3	57.0 (3)	C70—C69—C72	122.4 (6)
O1—C1—Sm3	63.1 (3)	C68—C69—C72	119.9 (6)
C2—C1—Sm3	169.5 (3)	C71—C70—C69	121.4 (6)
C3—C2—C7	119.0 (5)	C71—C70—H70	119.3
C3—C2—C1	118.8 (5)	C69—C70—H70	119.3
C7—C2—C1	122.2 (5)	C70—C71—C66	120.8 (5)
C2—C3—C4	120.0 (6)	C70—C71—H71	119.6
C2—C3—H3	120.0	C66—C71—H71	119.6
C4—C3—H3	120.0	C69—C72—H72A	109.5
C5—C4—C3	121.8 (6)	C69—C72—H72B	109.5
C5—C4—H4	119.1	H72A—C72—H72B	109.5
C3—C4—H4	119.1	C69—C72—H72C	109.5
C4—C5—C6	117.3 (6)	H72A—C72—H72C	109.5
C4—C5—C8	120.9 (7)	H72B—C72—H72C	109.5
C6—C5—C8	121.8 (7)	O5—Sm2—O4	83.57 (14)
C5—C6—C7	122.6 (6)	O5—Sm2—O10	79.94 (13)
C5—C6—H6	118.7	O4—Sm2—O10	150.81 (12)
C7—C6—H6	118.7	O5—Sm2—O19	91.39 (14)
C2—C7—C6	119.3 (6)	O4—Sm2—O19	83.42 (12)
C2—C7—H7	120.3	O10—Sm2—O19	73.13 (11)
C6—C7—H7	120.3	O5—Sm2—O14	91.41 (16)
C5—C8—H8A	109.5	O4—Sm2—O14	120.44 (12)
C5—C8—H8B	109.5	O10—Sm2—O14	84.06 (12)
H8A—C8—H8B	109.5	O19—Sm2—O14	156.14 (12)
C5—C8—H8C	109.5	O5—Sm2—O11	157.21 (14)
H8A—C8—H8C	109.5	O4—Sm2—O11	117.79 (13)
H8B—C8—H8C	109.5	O10—Sm2—O11	77.34 (12)
O3—C9—O4	119.7 (5)	O19—Sm2—O11	83.84 (14)
O3—C9—C10	121.6 (5)	O14—Sm2—O11	84.44 (17)
O4—C9—C10	118.7 (5)	O5—Sm2—O12	150.25 (13)
O3—C9—Sm3	58.3 (3)	O4—Sm2—O12	70.74 (12)
O4—C9—Sm3	61.8 (3)	O10—Sm2—O12	129.62 (12)
C10—C9—Sm3	173.5 (4)	O19—Sm2—O12	100.05 (12)
C15—C10—C11	117.4 (5)	O14—Sm2—O12	89.01 (14)
C15—C10—C9	122.8 (5)	O11—Sm2—O12	52.31 (12)
C11—C10—C9	119.7 (5)	O5—Sm2—O13	90.97 (13)
C12—C11—C10	120.4 (6)	O4—Sm2—O13	69.12 (11)
C12—C11—H11	119.8	O10—Sm2—O13	134.67 (11)
C10—C11—H11	119.8	O19—Sm2—O13	151.98 (11)
C11—C12—C13	121.3 (6)	O14—Sm2—O13	51.61 (12)
C11—C12—H12	119.4	O11—Sm2—O13	103.67 (13)
C13—C12—H12	119.4	O12—Sm2—O13	66.35 (11)
C14—C13—C12	118.1 (6)	O5—Sm2—C41	174.76 (15)
C14—C13—C16	121.7 (7)	O4—Sm2—C41	95.69 (14)
C12—C13—C16	120.1 (7)	O10—Sm2—C41	102.81 (14)
C15—C14—C13	121.0 (6)	O19—Sm2—C41	93.68 (14)
C15—C14—H14	119.5	O14—Sm2—C41	84.48 (16)
C13—C14—H14	119.5	O11—Sm2—C41	25.60 (13)
C14—C15—C10	121.8 (6)	O12—Sm2—C41	26.83 (13)
C14—C15—H15	119.1	O13—Sm2—C41	83.93 (13)
C10—C15—H15	119.1	O5—Sm2—C57	92.23 (15)
C13—C16—H16A	109.5	O4—Sm2—C57	95.44 (13)
C13—C16—H16B	109.5	O10—Sm2—C57	109.06 (13)
H16A—C16—H16B	109.5	O19—Sm2—C57	176.06 (13)
C13—C16—H16C	109.5	O14—Sm2—C57	25.25 (13)
H16A—C16—H16C	109.5	O11—Sm2—C57	93.40 (15)
H16B—C16—H16C	109.5	O12—Sm2—C57	76.02 (14)
O6—C17—O5	122.9 (5)	O13—Sm2—C57	26.38 (12)
O6—C17—C18	119.5 (5)	C41—Sm2—C57	82.67 (15)
O5—C17—C18	117.7 (5)	O5—Sm2—Sm3	110.45 (10)
C23—C18—C19	118.3 (5)	O4—Sm2—Sm3	42.28 (8)
C23—C18—C17	120.5 (5)	O10—Sm2—Sm3	166.45 (8)
C19—C18—C17	121.2 (5)	O19—Sm2—Sm3	114.11 (8)
C18—C19—C20	120.4 (6)	O14—Sm2—Sm3	86.94 (9)
C18—C19—H19	119.8	O11—Sm2—Sm3	91.74 (9)
C20—C19—H19	119.8	O12—Sm2—Sm3	39.86 (8)
C21—C20—C19	120.8 (7)	O13—Sm2—Sm3	39.90 (7)
C21—C20—H20	119.6	C41—Sm2—Sm3	66.16 (11)
C19—C20—H20	119.6	C57—Sm2—Sm3	63.07 (10)
C22—C21—C20	117.9 (6)	O5—Sm2—Sm1	69.64 (11)
C22—C21—C24	120.7 (7)	O4—Sm2—Sm1	111.92 (8)
C20—C21—C24	121.4 (7)	O10—Sm2—Sm1	39.41 (8)
C21—C22—C23	121.9 (6)	O19—Sm2—Sm1	39.00 (8)
C21—C22—H22	119.0	O14—Sm2—Sm1	121.50 (9)
C23—C22—H22	119.0	O11—Sm2—Sm1	93.49 (9)
C18—C23—C22	120.6 (6)	O12—Sm2—Sm1	133.55 (8)
C18—C23—H23	119.7	O13—Sm2—Sm1	159.97 (8)
C22—C23—H23	119.7	C41—Sm2—Sm1	115.32 (11)
C21—C24—H24A	109.5	C57—Sm2—Sm1	144.33 (10)
C21—C24—H24B	109.5	Sm3—Sm2—Sm1	151.439 (9)
H24A—C24—H24B	109.5	O15—Sm3—O13	82.56 (13)
C21—C24—H24C	109.5	O15—Sm3—O8^ii^	85.66 (14)
H24A—C24—H24C	109.5	O13—Sm3—O8^ii^	145.46 (12)
H24B—C24—H24C	109.5	O15—Sm3—O12	79.03 (14)
O8—C25—O7	119.3 (4)	O13—Sm3—O12	70.41 (12)
O8—C25—C26	119.4 (5)	O8^ii^—Sm3—O12	75.52 (12)
O7—C25—C26	121.3 (5)	O15—Sm3—O2	123.04 (14)
O8—C25—Sm3^i^	56.5 (3)	O13—Sm3—O2	78.08 (13)
O7—C25—Sm3^i^	62.9 (2)	O8^ii^—Sm3—O2	134.44 (14)
C26—C25—Sm3^i^	173.9 (3)	O12—Sm3—O2	138.73 (13)
C27—C26—C31	118.6 (5)	O15—Sm3—O3	158.87 (14)
C27—C26—C25	120.0 (5)	O13—Sm3—O3	117.52 (12)
C31—C26—C25	121.4 (5)	O8^ii^—Sm3—O3	73.96 (13)
C26—C27—C28	120.7 (6)	O12—Sm3—O3	100.68 (13)
C26—C27—H27	119.7	O2—Sm3—O3	70.89 (14)
C28—C27—H27	119.7	O15—Sm3—O4	142.51 (13)
C29—C28—C27	121.0 (6)	O13—Sm3—O4	69.06 (11)
C29—C28—H28	119.5	O8^ii^—Sm3—O4	103.83 (12)
C27—C28—H28	119.5	O12—Sm3—O4	68.86 (12)
C28—C29—C30	118.4 (5)	O2—Sm3—O4	75.45 (12)
C28—C29—C32	120.8 (6)	O3—Sm3—O4	51.48 (11)
C30—C29—C32	120.8 (6)	O15—Sm3—O7^ii^	80.64 (13)
C29—C30—C31	120.7 (6)	O13—Sm3—O7^ii^	154.26 (11)
C29—C30—H30	119.6	O8^ii^—Sm3—O7^ii^	51.94 (11)
C31—C30—H30	119.6	O12—Sm3—O7^ii^	124.60 (11)
C26—C31—C30	120.5 (5)	O2—Sm3—O7^ii^	95.00 (13)
C26—C31—H31	119.7	O3—Sm3—O7^ii^	82.37 (11)
C30—C31—H31	119.7	O4—Sm3—O7^ii^	133.68 (11)
C29—C32—H32A	109.5	O15—Sm3—O1	74.43 (13)
C29—C32—H32B	109.5	O13—Sm3—O1	87.99 (11)
H32A—C32—H32B	109.5	O8^ii^—Sm3—O1	119.77 (11)
C29—C32—H32C	109.5	O12—Sm3—O1	147.68 (12)
H32A—C32—H32C	109.5	O2—Sm3—O1	52.04 (12)
H32B—C32—H32C	109.5	O3—Sm3—O1	110.73 (12)
O9—C33—O10	119.1 (5)	O4—Sm3—O1	126.30 (11)
O9—C33—C34	121.5 (5)	O7^ii^—Sm3—O1	68.79 (10)
O10—C33—C34	119.2 (5)	O15—Sm3—C1	98.67 (15)
O9—C33—Sm1	57.9 (3)	O13—Sm3—C1	79.82 (13)
O10—C33—Sm1	66.0 (3)	O8^ii^—Sm3—C1	134.21 (13)
C34—C33—Sm1	153.8 (4)	O12—Sm3—C1	150.21 (13)
C35—C34—C39	119.0 (6)	O2—Sm3—C1	25.44 (13)
C35—C34—C33	119.0 (5)	O3—Sm3—C1	91.81 (14)
C39—C34—C33	121.9 (5)	O4—Sm3—C1	99.77 (14)
C34—C35—C36	120.6 (7)	O7^ii^—Sm3—C1	83.59 (12)
C34—C35—H35	119.7	O1—Sm3—C1	26.80 (13)
C36—C35—H35	119.7	O15—Sm3—C25^ii^	83.55 (14)
C35—C36—C37	121.0 (7)	O13—Sm3—C25^ii^	164.44 (13)
C35—C36—H36	119.5	O8^ii^—Sm3—C25^ii^	25.50 (13)
C37—C36—H36	119.5	O12—Sm3—C25^ii^	100.07 (14)
C38—C37—C36	117.7 (6)	O2—Sm3—C25^ii^	115.64 (15)
C38—C37—C40	121.4 (8)	O3—Sm3—C25^ii^	75.67 (13)
C36—C37—C40	120.9 (8)	O4—Sm3—C25^ii^	119.95 (12)
C39—C38—C37	121.8 (7)	O7^ii^—Sm3—C25^ii^	26.48 (12)
C39—C38—H38	119.1	O1—Sm3—C25^ii^	94.98 (13)
C37—C38—H38	119.1	C1—Sm3—C25^ii^	109.24 (14)
C38—C39—C34	119.9 (6)	O15—Sm3—C9	165.08 (15)
C38—C39—H39	120.1	O13—Sm3—C9	93.31 (13)
C34—C39—H39	120.1	O8^ii^—Sm3—C9	89.84 (14)
C37—C40—H40A	109.5	O12—Sm3—C9	86.07 (14)
C37—C40—H40B	109.5	O2—Sm3—C9	69.55 (14)
H40A—C40—H40B	109.5	O3—Sm3—C9	25.23 (13)
C37—C40—H40C	109.5	O4—Sm3—C9	26.35 (12)
H40A—C40—H40C	109.5	O7^ii^—Sm3—C9	107.60 (13)
H40B—C40—H40C	109.5	O1—Sm3—C9	119.91 (13)
O11—C41—O12	118.9 (5)	C1—Sm3—C9	94.67 (15)
O11—C41—C42	121.9 (5)	C25^ii^—Sm3—C9	98.37 (14)
O12—C41—C42	119.2 (5)	O16^i^—Sm1—O6	77.16 (15)
O11—C41—Sm2	58.0 (3)	O16^i^—Sm1—O7	97.47 (14)
O12—C41—Sm2	61.3 (3)	O6—Sm1—O7	80.80 (13)
C42—C41—Sm2	173.3 (4)	O16^i^—Sm1—O20	81.90 (16)
C47—C42—C43	118.4 (5)	O6—Sm1—O20	120.15 (15)
C47—C42—C41	122.0 (5)	O7—Sm1—O20	157.90 (12)
C43—C42—C41	119.6 (5)	O16^i^—Sm1—O1^i^	78.74 (14)
C44—C43—C42	120.3 (6)	O6—Sm1—O1^i^	142.47 (14)
C44—C43—H43	119.9	O7—Sm1—O1^i^	74.30 (11)
C42—C43—H43	119.9	O20—Sm1—O1^i^	83.98 (12)
C43—C44—C45	121.6 (6)	O16^i^—Sm1—O9	152.02 (13)
C43—C44—H44	119.2	O6—Sm1—O9	128.40 (13)
C45—C44—H44	119.2	O7—Sm1—O9	78.50 (13)
C44—C45—C46	118.2 (6)	O20—Sm1—O9	91.68 (15)
C44—C45—C48	121.6 (7)	O1^i^—Sm1—O9	73.50 (12)
C46—C45—C48	120.2 (7)	O16^i^—Sm1—O19	106.14 (14)
C47—C46—C45	120.6 (6)	O6—Sm1—O19	82.10 (14)
C47—C46—H46	119.7	O7—Sm1—O19	146.82 (11)
C45—C46—H46	119.7	O20—Sm1—O19	51.44 (11)
C42—C47—C46	120.9 (6)	O1^i^—Sm1—O19	132.51 (11)
C42—C47—H47	119.6	O9—Sm1—O19	90.38 (12)
C46—C47—H47	119.6	O16^i^—Sm1—O10	155.98 (13)
C45—C48—H48A	109.5	O6—Sm1—O10	79.20 (13)
C45—C48—H48B	109.5	O7—Sm1—O10	82.80 (11)
H48A—C48—H48B	109.5	O20—Sm1—O10	106.72 (12)
C45—C48—H48C	109.5	O1^i^—Sm1—O10	123.81 (11)
H48A—C48—H48C	109.5	O9—Sm1—O10	51.72 (11)
H48B—C48—H48C	109.5	O19—Sm1—O10	66.16 (10)
O16—C49—O15	122.8 (5)	O16^i^—Sm1—C33	170.75 (15)
O16—C49—C50	118.0 (5)	O6—Sm1—C33	102.65 (15)
O15—C49—C50	119.1 (5)	O7—Sm1—C33	73.46 (13)
C55—C50—C51	119.8 (5)	O20—Sm1—C33	105.77 (15)
C55—C50—C49	119.6 (5)	O1^i^—Sm1—C33	96.70 (14)
C51—C50—C49	120.5 (5)	O9—Sm1—C33	25.92 (13)
C50—C51—C52	118.3 (6)	O19—Sm1—C33	82.90 (13)
C50—C51—H51	120.8	O10—Sm1—C33	27.13 (12)
C52—C51—H51	120.8	O16^i^—Sm1—C65	94.67 (15)
C53—C52—C51	122.2 (6)	O6—Sm1—C65	102.50 (15)
C53—C52—H52	118.9	O7—Sm1—C65	167.85 (13)
C51—C52—H52	118.9	O20—Sm1—C65	25.20 (13)
C52—C53—C54	118.3 (6)	O1^i^—Sm1—C65	107.76 (13)
C52—C53—C56	120.7 (7)	O9—Sm1—C65	90.52 (14)
C54—C53—C56	121.0 (7)	O19—Sm1—C65	26.25 (12)
C53—C54—C55	121.3 (6)	O10—Sm1—C65	86.33 (12)
C53—C54—H54	119.4	C33—Sm1—C65	94.39 (14)
C55—C54—H54	119.4	O16^i^—Sm1—Sm3^i^	73.87 (11)
C54—C55—C50	120.1 (6)	O6—Sm1—Sm3^i^	105.54 (11)
C54—C55—H55	120.0	O7—Sm1—Sm3^i^	39.39 (8)
C50—C55—H55	120.0	O20—Sm1—Sm3^i^	121.18 (9)
C53—C56—H56A	109.5	O1^i^—Sm1—Sm3^i^	39.50 (8)
C53—C56—H56B	109.5	O9—Sm1—Sm3^i^	86.66 (9)
H56A—C56—H56B	109.5	O19—Sm1—Sm3^i^	172.01 (8)
C53—C56—H56C	109.5	O10—Sm1—Sm3^i^	117.04 (7)
H56A—C56—H56C	109.5	C33—Sm1—Sm3^i^	97.46 (10)
H56B—C56—H56C	109.5	C65—Sm1—Sm3^i^	146.22 (10)
O14—C57—O13	119.8 (5)	O16^i^—Sm1—Sm2	126.90 (11)
O14—C57—C58	120.2 (5)	O6—Sm1—Sm2	65.48 (11)
O13—C57—C58	120.0 (5)	O7—Sm1—Sm2	111.45 (8)
O14—C57—Sm2	55.1 (3)	O20—Sm1—Sm2	85.62 (9)
O13—C57—Sm2	64.8 (3)	O1^i^—Sm1—Sm2	150.46 (9)
C58—C57—Sm2	174.3 (4)	O9—Sm1—Sm2	79.27 (9)
C59—C58—C63	118.7 (5)	O19—Sm1—Sm2	35.38 (7)
C59—C58—C57	119.7 (5)	O10—Sm1—Sm2	35.40 (7)
C63—C58—C57	121.5 (5)	C33—Sm1—Sm2	59.90 (10)
C60—C59—C58	120.7 (6)	C65—Sm1—Sm2	60.73 (10)
C60—C59—H59	119.6	Sm3^i^—Sm1—Sm2	150.230 (8)
C58—C59—H59	119.6	C1—O1—Sm1^ii^	140.1 (3)
C59—C60—C61	120.6 (6)	C1—O1—Sm3	90.1 (3)
C59—C60—H60	119.7	Sm1^ii^—O1—Sm3	103.95 (12)
C61—C60—H60	119.7	C1—O2—Sm3	97.6 (3)
C62—C61—C60	118.8 (5)	C9—O3—Sm3	96.5 (3)
C62—C61—C64	120.5 (6)	C9—O4—Sm2	160.7 (3)
C60—C61—C64	120.7 (6)	C9—O4—Sm3	91.9 (3)
C63—C62—C61	121.4 (6)	Sm2—O4—Sm3	99.26 (12)
C63—C62—H62	119.3	C17—O5—Sm2	137.5 (4)
C61—C62—H62	119.3	C17—O6—Sm1	142.2 (4)
C62—C63—C58	119.7 (5)	C25—O7—Sm1	145.9 (3)
C62—C63—H63	120.2	C25—O7—Sm3^i^	90.6 (3)
C58—C63—H63	120.2	Sm1—O7—Sm3^i^	104.23 (11)
C61—C64—H64A	109.5	C25—O8—Sm3^i^	98.0 (3)
C61—C64—H64B	109.5	C33—O9—Sm1	96.2 (3)
H64A—C64—H64B	109.5	C33—O10—Sm2	146.3 (3)
C61—C64—H64C	109.5	C33—O10—Sm1	86.8 (3)
H64A—C64—H64C	109.5	Sm2—O10—Sm1	105.19 (12)
H64B—C64—H64C	109.5	C41—O11—Sm2	96.4 (3)
O20—C65—O19	118.1 (4)	C41—O12—Sm3	162.5 (4)
O20—C65—C66	120.6 (5)	C41—O12—Sm2	91.9 (3)
O19—C65—C66	121.2 (4)	Sm3—O12—Sm2	98.76 (12)
O20—C65—Sm1	54.8 (3)	C57—O13—Sm3	145.0 (3)
O19—C65—Sm1	63.4 (2)	C57—O13—Sm2	88.8 (3)
C66—C65—Sm1	174.9 (4)	Sm3—O13—Sm2	96.52 (11)
C71—C66—C67	117.6 (5)	C57—O14—Sm2	99.7 (3)
C71—C66—C65	122.0 (5)	C49—O15—Sm3	146.8 (4)
C67—C66—C65	120.3 (5)	C49—O16—Sm1^ii^	129.5 (4)
C68—C67—C66	121.2 (6)	C65—O19—Sm2	155.4 (3)
C68—C67—H67	119.4	C65—O19—Sm1	90.4 (3)
C66—C67—H67	119.4	Sm2—O19—Sm1	105.62 (12)
C67—C68—C69	121.2 (6)	C65—O20—Sm1	100.0 (3)

Symmetry codes: (i) −*x*+1/2, *y*+1/2, −*z*+1/2; (ii) −*x*+1/2, *y*−1/2, −*z*+1/2.
